# Cardiac Effects of Attenuating Gsα - Dependent Signaling

**DOI:** 10.1371/journal.pone.0146988

**Published:** 2016-01-26

**Authors:** Marcus R. Streit, Celine S. Weiss, Sören Meyer, Marco M. Ochs, Marco Hagenmueller, Johannes H. Riffel, Sebastian J. Buss, Thomas Heger, Hugo A. Katus, Stefan E. Hardt

**Affiliations:** 1 Department of Cardiology, Heidelberg University Hospital, Im Neuenheimer Feld 410, 69120 Heidelberg, Germany; 2 Clinic for Orthopedics and Trauma Surgery, Center for Orthopedics, Trauma Surgery and Spinal Cord Injury, Heidelberg University Hospital, Schlierbacher Landstrasse 200a, 69118 Heidelberg, Germany; 3 Center for Cardiac and Circulatory Diseases, Hoheneggerstr. 9, 76646 Bruchsal, Germany; San Diego State University, UNITED STATES

## Abstract

**Aims:**

Inhibition of β-adrenergic signalling plays a key role in treatment of heart failure. Gsα is essential for β-adrenergic signal transduction. In order to reduce side-effects of beta-adrenergic inhibition diminishing β-adrenergic signalling in the heart at the level of Gsα is a promising option.

**Methods and Results:**

We analyzed the influence of Gsα on regulation of myocardial function and development of cardiac hypertrophy, using a transgenic mouse model (C57BL6/J mice) overexpressing a dominant negative Gsα-mutant under control of the α-MHC-promotor. Cardiac phenotype was characterized *in vivo* and *in vitro* and under acute and chronic β-adrenergic stimulation. At rest, Gsα-DN-mice showed bradycardia (602 ± 13 vs. 660 ± 17 bpm, *p*<0.05) and decreased dp/dt_max_ (5037 ± 546- vs. 6835 ± 505 mmHg/s, p = 0.02). No significant differences were found regarding ejection fraction, heart weight and cardiomyocyte size. β-blockade by propranolol revealed no baseline differences of hemodynamic parameters between wildtype and Gsα-DN-mice. Acute adrenergic stimulation resulted in decreased β-adrenergic responsiveness in Gsα-DN-mice. Under chronic adrenergic stimulation, wildtype mice developed myocardial hypertrophy associated with increase of LV/BW-ratio by 23% (4.4 ± 0.2 vs. 3.5 ± 0.1 mg/g, *p<0*.*01*) and cardiac myocyte size by 24% (14927 ± 442 px vs. 12013 ± 583 px, *p<0*.*001*). In contrast, both parameters were unchanged in Gsα-DN-mice after chronic isoproterenol stimulation.

**Conclusion:**

Overexpression of a dominant negative mutant of Gsα leads to decreased β-adrenergic responsiveness and is protective against isoproterenol-induced hypertrophy. Thus, Gsα-DN-mice provide novel insights into β-adrenergic signal transduction and its modulation in myocardial overload and failure.

## Introduction

While initially compensatory, in the long term, left ventricular remodeling frequently is a maladaptive process, characterized by myocyte hypertrophy, increase in myocardial fibrosis and left ventricular dilatation. This process may progress to clinically overt heart failure which contributes to increased mortality. Myocardial stress activates a number of signaling pathways which contribute to the remodeling process. The prominent role of the β-adrenergic signaling [1] and the discovery of the consistently beneficial effects of anti-β-adrenergic signalling therapy in CHF have greatly improved treatment in clinical practice [2]. By now, treatment with β-blockers is standard of therapy for patients with chronic heart failure due to left ventricular systolic dysfunction, with proven effects on mortality and symptoms related to worsening heart failure [2–6]. Despite the marked benefits of the actual mode of therapy there is still room for improvement since mortality and morbidity remain high for patients with heart failure. Although some people survive many years, progressive disease is associated with an overall annual mortality rate of 10% despite guidelines adjusted therapies [7]. In order to gain insights into potential novel therapies investigations about the mechanisms underlying distal components of β-adrenergic signaling involving are necessary.

The deleterious effects of chronic β-adrenergic receptor (AR) stimulation in the heart have been documented in several mouse models and in vivo [8]. As the use of β-blockade has been proven to be of benefit in clinical practice, it is conceivable that interrupting distal components in the β-adrenergic receptor-G protein-adenylyl cyclase pathway may also provide targets for future therapeutic modalities for heart failure. However, to date, only two animal models have been described, in which stimulating components of β-adrenergic signaling were inhibited downstream the receptor [9, 10].

A possible target downstream of the β1-AR is the stimulatory heterotrimeric guanyl nucleotide binding protein (Gs) and especially its α subunit (Gsα), which promotes exchange of GTP to GDP and for this reason is critical for signal transmission. Previous studies suggest a regulatory function in failing heart exceeding the "classical" β adrenergic signal transduction *[11, 12]*. Furthermore, recent investigations have revealed a novel, distinct cardioprotective set of β1-AR signals. These signals are carried G protein-independently by β-arrestins [13]. Bias between G proteins and β-arrestins offers a general strategy for beneficially separating β1-AR responses to achieve functional selectivity [14]. Currently, there is no strongly biased ligand at the β1-AR that antagonizes G protein mediated cAMP generation while promoting β-arrestin dependent signaling.

To learn more about Gsα´s role in cardiac contractility modulation and remodeling processes we attempted to delete Gsα in a transgenic mouse model. As homozygous Gsα knockout mice are embryonically lethal and also heterozygotes are not reaching adulthood [15] we decided to create a transgenic mouse model with a cardiac selective overexpression of a dominant negative Gsα mutant. For this mutant a selective inhibition of receptor-mediated stimulation of adenylyl cyclase is reported reducing cAMP accumulation up to ~80% in COS-7 cells [16].

## Materials and Methods

### Generation of transgenic mice

A cDNA encoding dominant-negative Gsα (Gsα-DN) mutant was cloned into a plasmid containing the heart-specific α-myosin heavy chain (MHC) promoter and human growth hormone (hGH) poly(A)^+^ signal. Corresponding cDNA sequence for Gsα triple mutant (G^226^A, E^268^A, A^366^S) was consistent with description from Iiri and colleagues [16]. DNA isolation and linearization were carried out using standard techniques. DNA injections into pronuclei of mice with B6D2 F1/Crl background were performed in the transgenic core facility of the University of Heidelberg, Germany. Genomic DNA was isolated from mouse tail biopsies and analyzed by PCR with primers specific for the hGH poly(A)^+^ signal: (forward: 5’- GTC TAT TCG GGA ACC AAG CTG GAG TG-3’, reverse: 5’- ACA GGC ATC TAC TGA GTG GAC CCA AC-3’). Cardiac-specific overexpression of Gsα-DN was affirmed by Western blotting. Animals were backcrossed with C57BL6/J (Charles River), starting the experiments not before 6^th^ generation to carry out all experiments in a C57BL6/J background. All animals analyzed were males at the age of 3 months (unless stated otherwise). Generation of transgenic animals as well as animal handling and experiments were performed according to the institutional guidelines of the University of Heidelberg and the Directive 2010/63/EU of the European Parliament. They were approved by the animal experiment review board of the government of the state of Baden-Württemberg, Germany (approval number: 35–9185.81/G-138/06). All surgery was performed under isoflurane anaesthesia, and all efforts were made to minimize suffering. For postoperative analgesia buprenorphine 0.05 mg/kg was administered subcutaneously. For echocardiography and during prearrangement for heart rate measurement, mice were sedated by isoflurane inhalation. The condition of the animals in all experimental lines was monitored daily. The animals observed to determine lifespan were allowed to live until natural death. However, they were euthanized when they reached a moribund state. The rest of the animals was sacrificed after in vivo measurements by arresting the heart in end-diastole by injection of 1ml of 1 mol/l KCl (B. Braun, Melsungen, Germany) under isoflurane anaesthesia.

### cAMP-EIA

Intracellular cyclic AMP (cAMP) levels were determined with a commercially available competitive binding immunoassay kit (Cyclic-AMP Enzyme Immunoassay Kit, Assay Designs, Ann Arbor, Michigan, USA). Under endotracheal anesthesia with 2% isoflurane (Isoflurane CP, CP-Pharma, Burgdorf, Germany) thoracotomy was performed and hearts were arrested in end-diastole by direct intraventricular injection of 1ml of 1 mol/l KCl at 4°C (B. Braun, Melsungen, Germany) and left ventricles were isolated followed by flash freezing in liquid nitrogen. Samples were dissolved in the accordant buffer and measurements were performed according to instruction of the manufacturer. cAMP concentration was determined by using a photometer at 405 nm wavelength. Resulting values of cAMP concentration were corrected for dilution effects.

### Mortality

To determine lifespan, a sub-group of wild type and transgenic animals was observed in a prospective study up to natural death (n = 65 for transgenic and n = 10 for wild type mice). These animals were not included in interventions or other investigations. Mice were euthanized using humane endpoints to prevent unnecessary suffering when they were moribund. Repeated ECGs and echocardiograms were done until the age of 20 months to detect age-related alterations of cardiac phenotype. After death, organs were excised and subjected to further analyses.

### Heart rate in conscious mice

For detecting heart rate in conscious mice, gel-coated ECG electrodes were fixed at the animals paws and the animal was brought in a measuring construction where it was not able to disconnect itself. To reduce stress reaction during prearrangement, animals were sedated short term with 3% isoflurane and then permitted to acclimatize themselves for 15 minutes prior to collection of baseline data. Using a 6-canal-ECG recorder (Cardioscript CD 6000; Picker-Schwarzer, Germany) signals were printed and simultaneously digitized by using PowerLab Chart Software Version 5.3 (ADInstruments Pty Ltd., Colorado Springs, CO, USA). Only data from continuous recordings of 20–30 ECG signals were used in the analyses. Signals were analyzed using PowerLab Chart Software Version 5.3 (ADInstruments Pty Ltd., Colorado Springs, CO, USA) with manual control in printed ECG records.

### Echocardiography

Transthoracic echocardiography was performed as previously described in detail [17]. Briefly, mice were sedated by isofluorane inhalation (2% isofluorane) and were allowed to breathe spontaneously. The chest was shaved, and mice were positioned on a warming pad (Fine Science Tools GmbH, Heidelberg, Germany) to keep temperature constant at 37°C. Echocardiography was performed by using an HDI 5000 CV echocardiography machine (ATL Ultrasound, Philips, Bothell, WA, USA) equipped with a 10-MHz probe. The heart was imaged using the two-dimensional mode in the parasternal short-axis view, including papillary muscles, to position the M-mode cursor perpendicular to the ventricular septum and LV posterior wall. Digital images were analysed by using a HDI Lab (ATL, Philips, Bothell, WA, USA). Measurements were performed using Scion Image for Windows (Scion Corp). M-mode measurements of left ventricular dimensions were averaged from at least three cycles, using the leading edge—to—leading edge convention adopted by the American Society of Echocardiography. The investigator who conducted the echocardiography was blinded for the treatment status.

### Left ventricular pressure volume measurements

Invasive assessment of hemodynamics was performed under endotracheal anesthesia with isoflurane (initially 5%, after intubation 2% isoflurane). Mice were placed on a heating pad and mechanically ventilated. The right internal carotid artery was exposed, and a microtip catheter transducer (SPR-839, Millar Instruments, Houston, Tex, USA) was inserted into the right carotid artery and advanced into the left ventricle under pressure control. After stabilization, the pressure signals were recorded continuously with a pressure volume conductance system (MPVS-300, Millar Instruments, Houston, Texas, USA) coupled with a PowerLab converter (PowerLab 4/20, ADInstru-ments Pty Ltd., Colorado Springs, CO, USA), stored by using suitable software (LabChart, ADInstruments), and displayed on a personal computer. PVAN software (Millar Instruments) was used for subsequent analysis of pressure-volume loops. The raw conductance volumes were corrected for parallel conductance by the hypertonic saline dilution method. For absolute volume measurements, the catheter was calibrated with known volumes of heparin-treated mouse blood.

### Acute treatment with β adrenergic stimulator

A group of transgenic and wild type mice was assigned to a isoproterenol-treated group (TG n = 12; WT n = 13). Isoproterenol HCl (I6504, Sigma-Aldrich, St. Louis, MO), which is a synthetic catecholamine that stimulates both β1 and β2 adrenergic receptors (no alpha receptor capabilities), was administered intravenous by catheterization of the internal jugular vein at increasing concentrations (0,02 μg/kg/min. → 0,05 μg/kg/min. → 0,1 μg/kg/min. → 0,2 μg/kg/min.; with step-up every 3 minutes). Then, all animal were treated with intravenous propranolol HCl (P0884, Sigma-Aldrich, St. Louis, MO) as a nonselective β AR blocker at a dosis of 1 mg/kg/min for 2 minutes.

To evaluate the extent of maximal β-adrenergic stimulation, additional transgenic animals (n = 7) were exposed to higher doses of isoproterenol (0,2 μg/kg/min. → 0,5 μg/kg/min. → 1,0 μg/kg/min. → 1,5 μg/kg/min. → 2,0 μg/kg/min.; with step-up every 3 minutes). This was carried out in an additional experimental setup to avoid the influence of volume overload.

### Histopathology

After in vivo hemodynamic measurements, the heart was arrested in end-diastole by injection of 1ml of 1 mol/l KCl (B. Braun, Melsungen, Germany) under isoflurane anaesthesia. Organs such as heart, lung and liver were excised, weighed, and frozen in liquid nitrogen. The heart was divided into the atria, left ventricle including the intraventricular septum, and the right ventricle. LV/body weight ratio, heart/body weight ratio, lung/body weight ratio and liver/body weight ratio were determined. Histological studies were conducted using formalin-fixed, paraffin embedded hearts from animals of all groups. Cross sections of the LV obtained midway between base and apex were stained with hematoxylin/eosin and myocyte size was measured as cross sectional area using the ImageJ software (ImageJ, NIH, USA). Additionally, picrosirius red staining was done to detect collagen deposition. Evidence of fibrosis was evaluated using the ImageJ software (ImageJ, NIH, USA) in light microscopy pictures. Collagen area fraction in percent was then calculated as collagen area to tissue area ratio.

### Immunoblot analyses

Protein lysates were prepared from the left ventricles of WT and transgenic mice (3 months unless otherwise stated). Protein concentration was measured using BCA protein assay (Interchim). Equal amounts of protein were separated with SDS-PAGE and transferred to a nitrocellulose membrane (Millipore). The membranes were incubated overnight at 4°C with primary antibody. Antibodies used were *anti-Gsα (K-20*, *Santa Cruz*, *sc-823)*, *anti-Galpha i/o/t/z (D-15*, *Santa Cruz*, *sc-12798)*, *anti-Serca 2ATPase (Thermo Scientific*, *MA3-919) and anti-alpha-sarcomeric actin*, *clone 5C5 (Sigma-Aldrich*, *A2172)*, Anti-G protein alpha inhibitor 1 [EPR9441(B)] (Abcam, ab140125), Anti-beta 1 Adrenergic Receptor antibody (Abcam, ab3442), β2-AR Antibody (H-20) (Santa-cruz, sc-569) and Anti-GAPDH antibody—loading control (Abcam, ab9485). Anti-rabbit IgG and anti-mouse IgG horseradish peroxidase-conjugated antibodies (Cell Signaling Technology) and Goat Anti-Rabbit IgG H&L (HRP) (Abcam, ab6721) were used as secondary antibodies. GAPDH was used as an internal control. Protein bands were subsequently detected with enhanced chemiluminescence and sections were exposed to X-ray film. Images were captured using a Peqlab Fusion FX imaging system (Peqlab Inc., Erlangen, Germany) and AlphaView software (ProteinSimple Inc., Santa Clara, CA) was used to quantify protein band densities.

### Quantitative real-time PCR

Total RNA was isolated from mouse left ventricular tissue using the Trizol reagent (Invitrogen). cDNA was synthesized with Revert Aid First strand cDNA synthesis kit (Fermentas). Real-time polymerase chain reaction (PCR) was performed using the LightCycler^®^ system (Roche Diagnostics, Mannheim, Germany) according to the manufacturer´s instructions. All real-time PCR sample reactions were performed in triplicate and normalized to HPRT mRNA expression. Primers and specific probes were designed using the Universal Probe Library from Roche Diagnostics. The following primers were used: for HPRT 5′-GTCAAGGGGGACATAAAAG-3′ and 5′-TGCATTGTTTTACCAGTGTCAA-3′, probe # 22; for atrial natriuretic factor (ANF) 5′-CAACACAGATCTGATGGATTTCA-3′ and 5′-CCTCATCTTCTACCGGCATC-3′, probe # 25. A standard curve was run with the dilution series of the amplified fragment allowing for mRNA copy number calculation.

### Chronic β adrenergic stimulation via Isoproterenol

Mice were anesthetized with isoflurane, and Alzet osmotic minipumps (model 2002, Durect Corp, Cupertino, CA) were implanted subcutaneously in the neck. For postoperative analgesia buprenorphine 0.05 mg/kg was administered subcutaneously. Mice were randomized to receive isoproterenol HCl (I6504, Sigma-Aldrich, St. Louis, MO) at a dose of 30 μg/g per day, dissolved in acidified isotonic saline, or isovolumic acidified saline alone (vehicle). Isoproterenol treatment was continued for 14 days.

### Statistical analysis

Data are reported as mean ± SEM. Differences between groups were tested for statistical significance using two-tailed Student t-test or multiple analysis of variance, when applicable, with Bonferroni post-hoc tests. SPSS V15.0 and Graphpad Prism V5.01 were used for statistical analysis. A *P*-value of <0.05 was considered significant. Error bars in figures indicate standard error if not indicated otherwise.

## Results

### Baseline characteristics of Gsα-DN-mice

**Fig 1:**
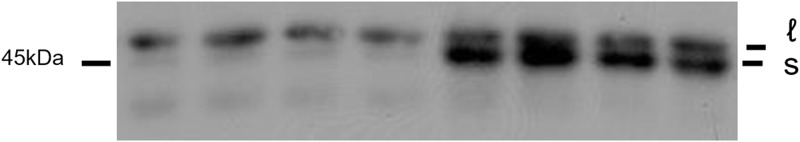
Confirmation of the overexpression of Gsα in transgenic mice at the protein level by immunoblot analysis. ℓ marks the long isoform, s the short isoform of Gsα Protein, which was overexpressed in this study.

To study the role of Gsα—dependent signaling in the postnatal heart we generated mice with cardiac specific overexpression of a dominant negative Gsα-mutant under the control of α-MHC promoter. Overexpression in transgenic Gsα-DN-mice was confirmed in protein analysis of left ventricular tissue by western blotting (Fig 1). In contrast to Gsα-knockout- (Gsα-KO-) mice which show an embryonic lethal phenotype or heterozygous Gsα +/- –mice which do not reach adulthood [15], our transgenic Gsα-DN-mice were both viable and fertile showing no increased mortality.

**Fig 2:**
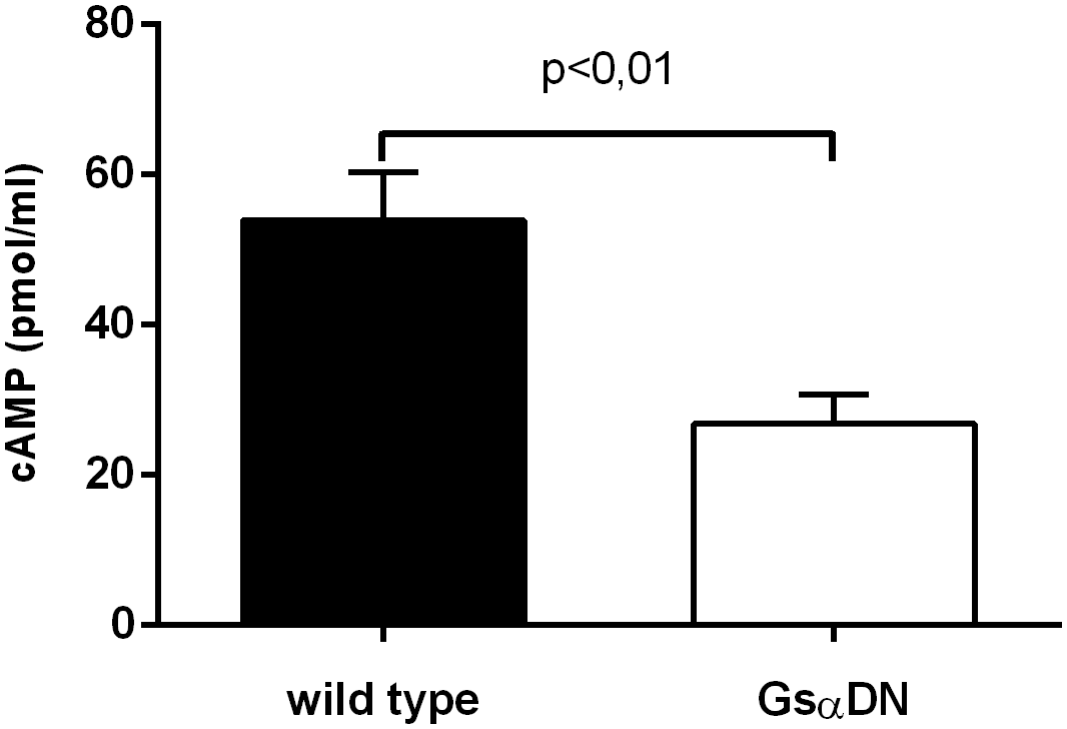
Concentrations of intracellular cAMP levels in left ventricular tissue in wild type and Gsα-DN-mice (n = 8 for wild type and n = 7 for transgenic animals).

**Fig 3:**
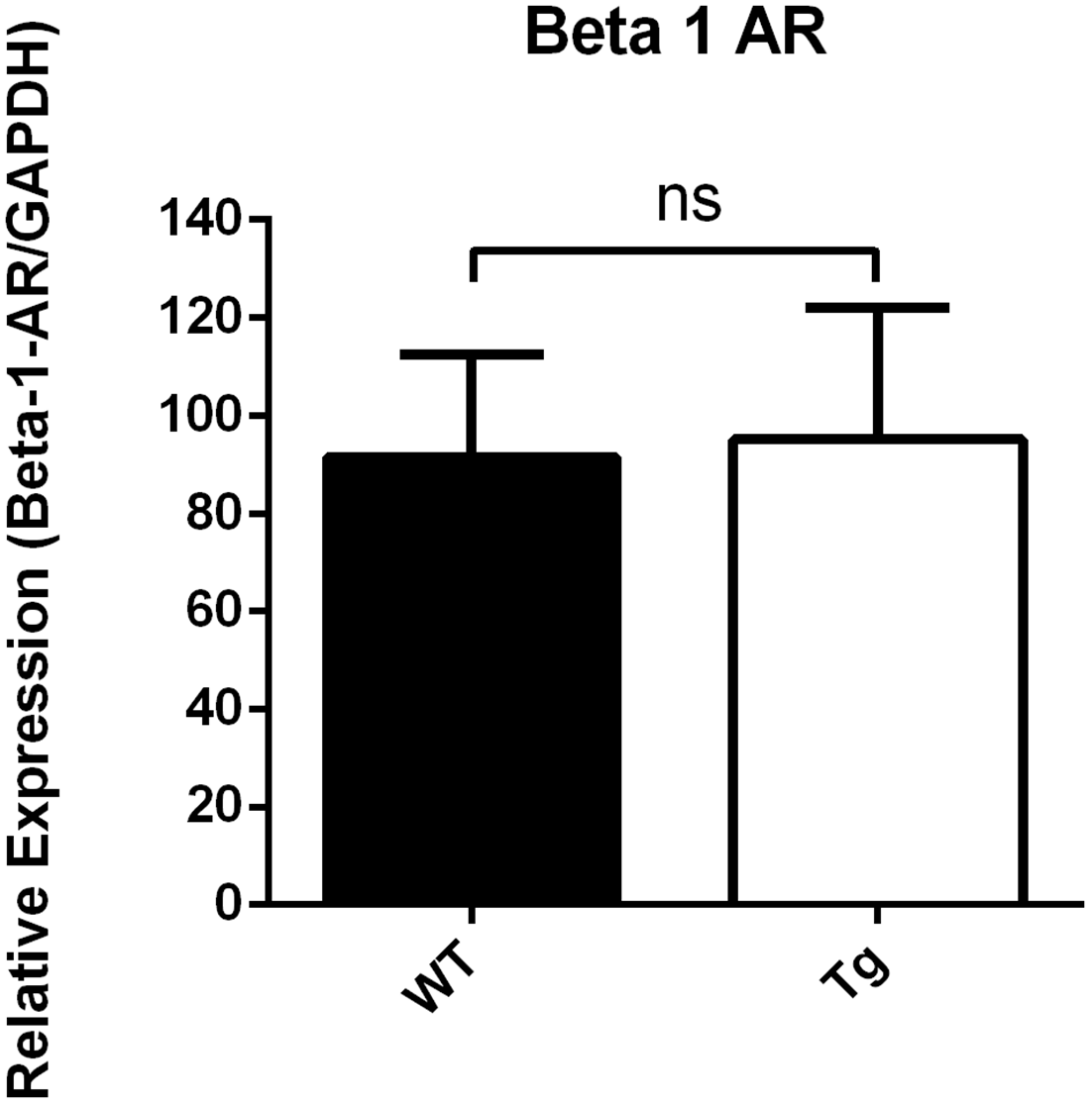
Western blot analysis of protein expression levels of β1- AR in the left ventricle (Gsα-DN-mice compared to wild types).

**Fig 4:**
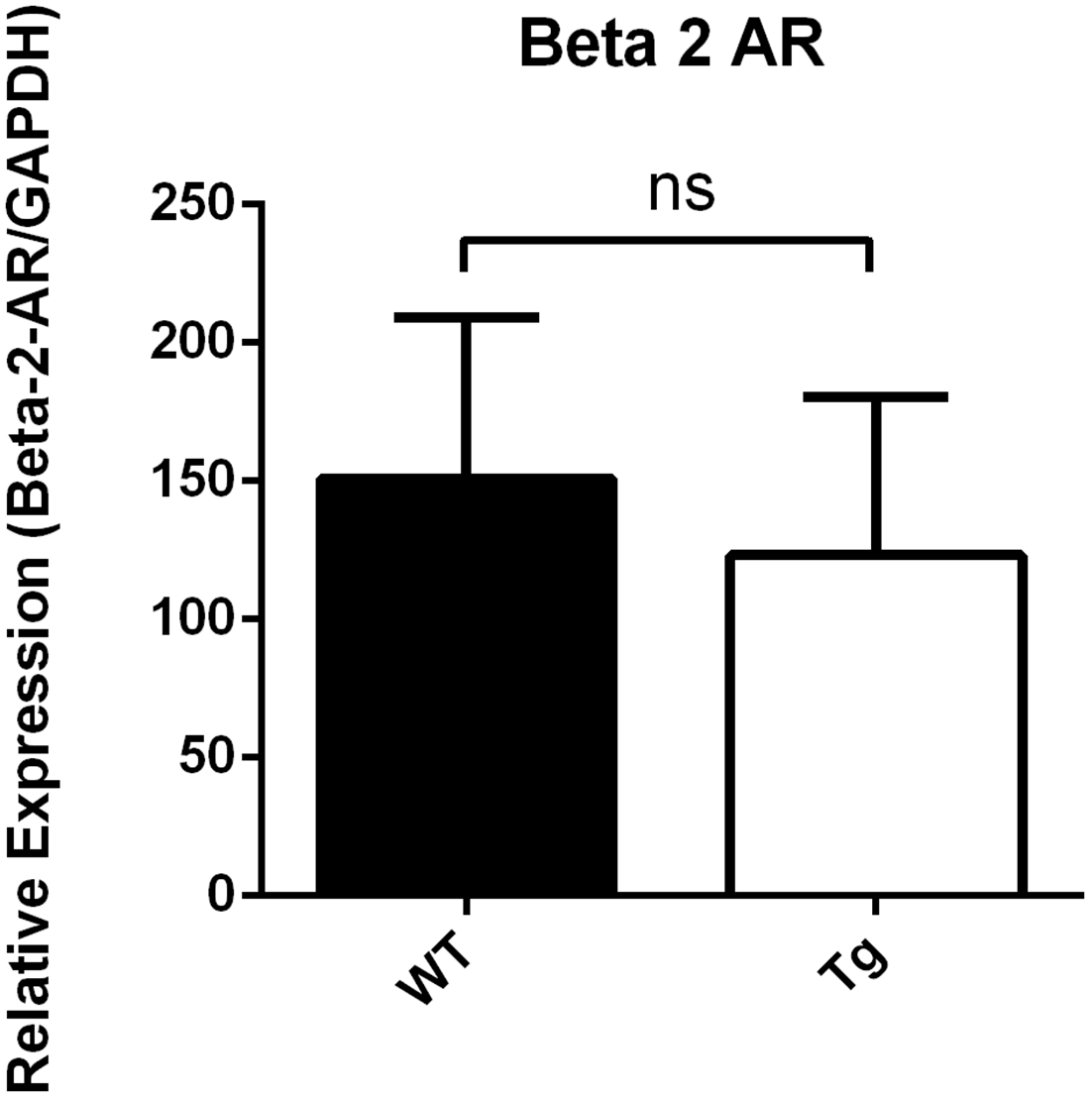
Western blot analysis of protein expression levels of β2- AR in the left ventricle (Gsα-DN-mice compared to wild types).

Analyses of intracellular cAMP levels of left ventricular tissue with a competitive binding immunoassay kit showed a cAMP reduction in transgenic Gsα-DN-mice of 50% in comparison to wild type (27 ± 4 vs. 54 ± 6 pmol/ml, p = 0.0041) (Fig 2). Expression of atrial natriuretic peptide (ANF) on mRNA-level as hypertrophic marker differed not significantly in Gsα-DN-mice compared to wild types at the age of 3 months (ANF/HPRT 3.53 ± 1.1 vs. 3.52 ± 1.0 normalized ratio). Western blot analysis showed no significant difference in protein expression levels of β1- and β2- AR in the left ventricle between Gsα-DN-mice compared to wild types (p = 0.25 and p = 0.99, respectively; Figs 3 and 4).

**Fig 5:**
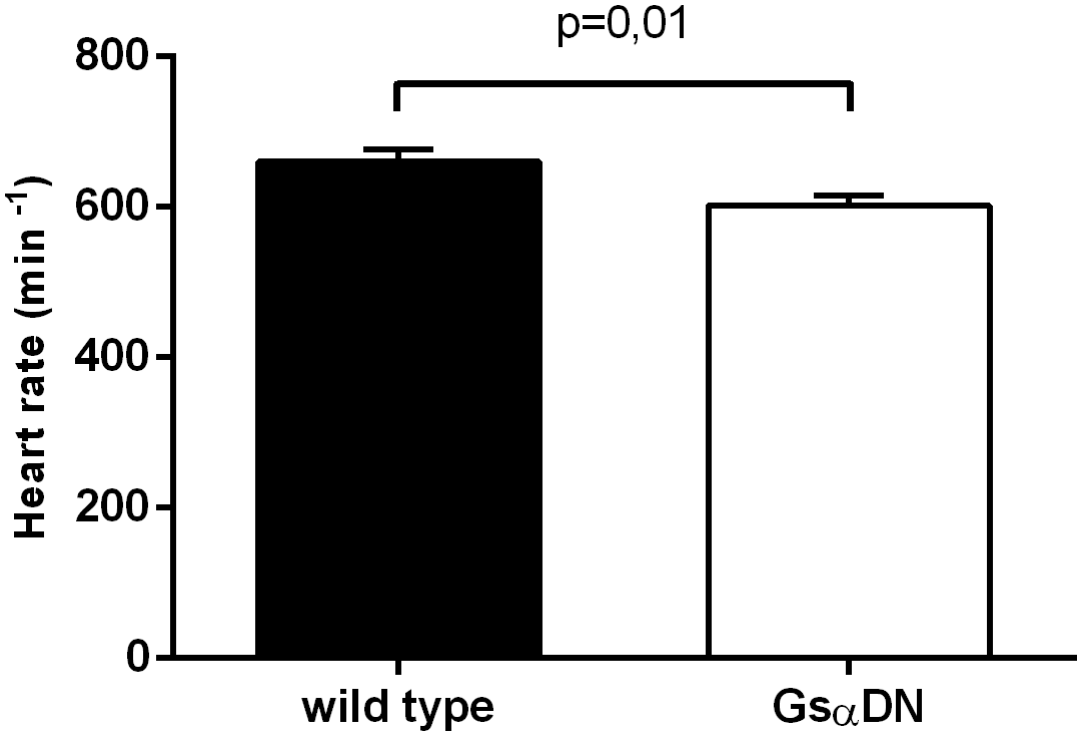
Heart rate in conscious mice is significantly reduced in Gsα-DN-mice in comparison to wild type (n = 14 for wild type and n = 13 for Gsα-DN-mice).

**Fig 6:**
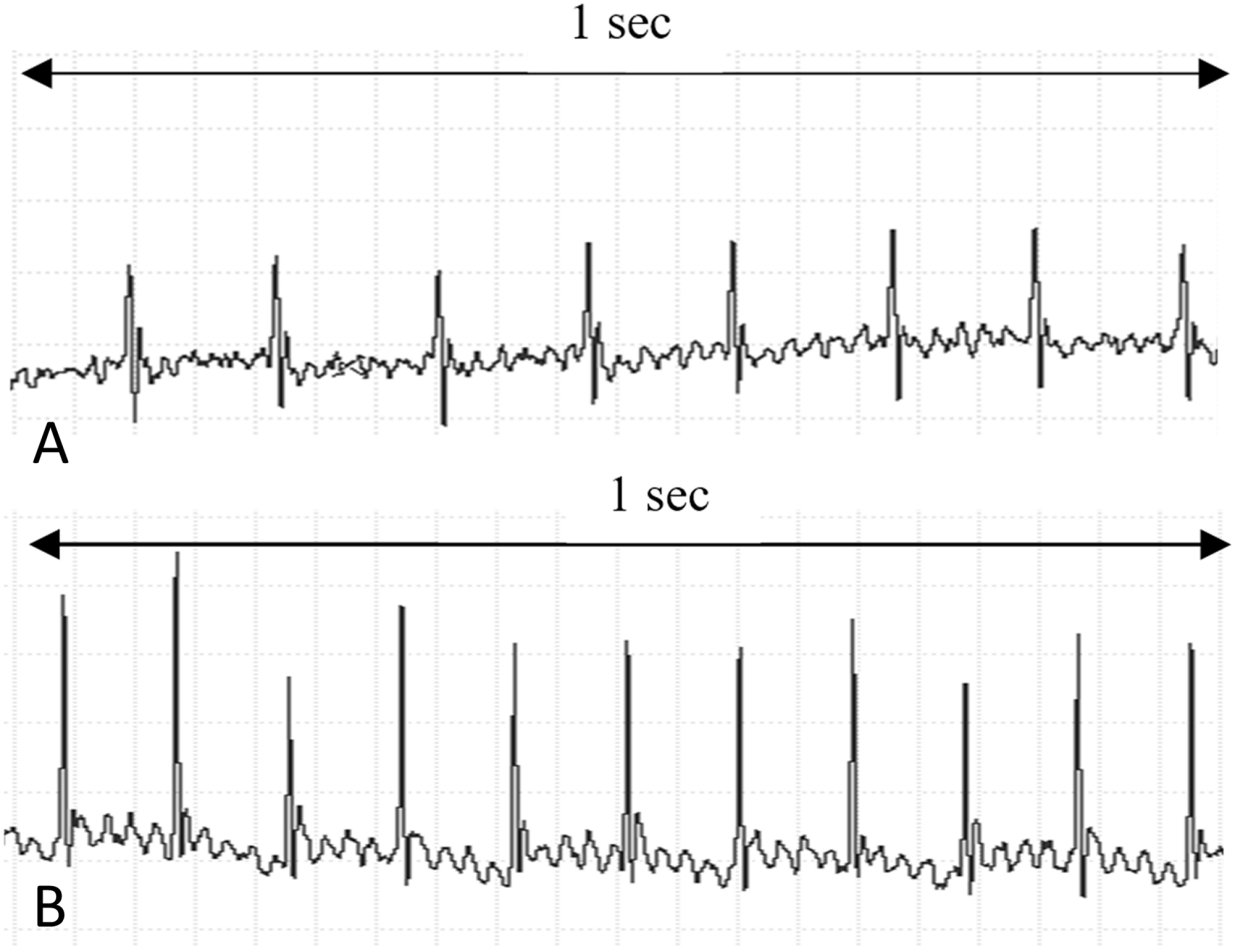
Heart rate in conscious mice at the age of 3 months. **(A)** Exemplary detail of a Gsα-DN-mouse ECG **(B)** Exemplary detail of a wild type ECG.

At rest, conscious Gsα-DN-mice showed a significant lower heart rate in comparison to wild type littermates (602 ± 13 vs. 660 ± 17 bpm at the age of 3 months, *p* = 0.01; Fig 5). ECGs showed no other abnormalities in transgenic animals, especially atrioventricular conduction delay or the occurrence of bundle branch blocks were not increased (Fig 6).

Regarding gross morphological parameters of hypertrophy such as heart to body weight ratio, LV to body weight ratio and also cardiomyocyte size, no significant differences were found (Table 1). Organ weights revealed no signs for cardiac decompensation as well. Also regarding cardiac fibrosis, there were no significant differences in the extent of collagen deposition in wild type and transgenic animals (3.7 ± 0.4 vs. 3.5 ± 0.5% collagen area fraction at the age of 3 months, n.s.).

**Table 1:** Baseline characterisation at the age of 3 months: Morphological parameters.

	Heart/body weight ratio [mg/g]	LV/body weight ratio [mg/g]	RV/body weight ratio [mg/g]	Lung/body weight ratio [mg/g]	Liver/body weight ratio [mg/g]	Myocyte size [px]
**Wild type** n = 27	4.5 ± 0.1	3.4 ± 0.08	0.8 ± 0.03	4.9 ± 0.24	45.1 ± 1.4	12014 ± 583
**Gsα-DN** n = 15	4.8 ± 0.1	3.6 ± 0.1	0.9 ± 0.05	4.6 ± 0.3	48.9 ± 2.3	11494 ±489
***p-value***	*n*.*s*.	*n*.*s*.	*n*.*s*.	*n*.*s*.	*n*.*s*.	*n*.*s*.

Echocardiographic studies confirmed bradycardia in sedated Gsα-DN-mice in comparison to wild type animals (447 ± 14 bpm vs. 501 ± 17 bpm, *p*<0.05). Apart from this there were no significant differences in echocardiographic values, especially not for end-diastolic diameter (EDD) or ejection fraction (EF) between both groups (Table 2). In invasive pressure-volume measurements, Gsα-DN-mice showed decreased dp/dt_max_ at rest. There was a trend toward lower dp/dt_min_ in Gsα-DN-mice, while left ventricular relaxation time constant (Tau_w_) was significantly reduced in Gsα-DN-mice. In deeper anesthetized animals, invasive measurements reconfirmed a trend toward lower heart rate in Gsα-DN-mice though not reaching statistical significance (530 ± 15 bpm vs. 488 ± 16 bpm, *p* = 0.07). No differences were found regarding other pressure-volume parameters. Hemodynamic data at baseline are shown in Table 3.

**Table 2:** Baseline characterisation at the age of 3 months: Echocardiographic parameters.

	Heart rate [bpm]	AWTD [mm]	EDD [mm]	PWTD [mm]	EF [%]	FS [%]
**Wild type** n = 11	501 ± 17	0.4 ± 0.01	4.6 ± 0.1	0.4 ± 0.01	75 ± 1	38 ± 1
**Gsα-DN** n = 11	447 ± 14	0.4 ± 0.01	4.7 ± 0.1	0.4 ± 0.01	73 ± 1	36 ± 1
***p-value***	*p<0*.*05*	*n*.*s*.	*n*.*s*.	*n*.*s*.	*n*.*s*.	*n*.*s*.

AWTD = anterior wall thickness diameter; EDD = end-diastolic diameter; PWTD = posterior wall thickness diameter; EF = ejection fraction; FS = fractional shortening.

**Table 3:** Baseline characterisation at the age of 3 months: Hemodynamic measurements.

	Heart rate [bmp]	LVEDP [mmHg]	LVESP [mmHg]	Stroke volume [μl]	EF [%]	dp/dt_max_ [mmHg/s]	dp/dt_min_ [mmHg/s]	Tau_w_ [ms]
**Wild type** n = 16	530 ± 15	3.8 ± 0.2	73.0 ± 3	24.3 ± 3.9	62 ± 3	6835 ± 505	-7290 ± 452	5.0 ± 0.2
**Gsα-DN** n = 13	488 ± 16	3.9 ± 0.9	69.2 ± 3	17.9 ± 1.9	57 ± 3	5037 ± 546	-6249 ± 692	6.5 ± 0.6
***p-value***	*n*.*s*.	*n*.*s*.	*n*.*s*.	*n*.*s*.	*n*.*s*.	*p = 0*.*02*	*n*.*s*.	*p = 0*.*02*

### Gsα-DN-mice reveal preserved left ventricular function with aging

**Fig 7:**
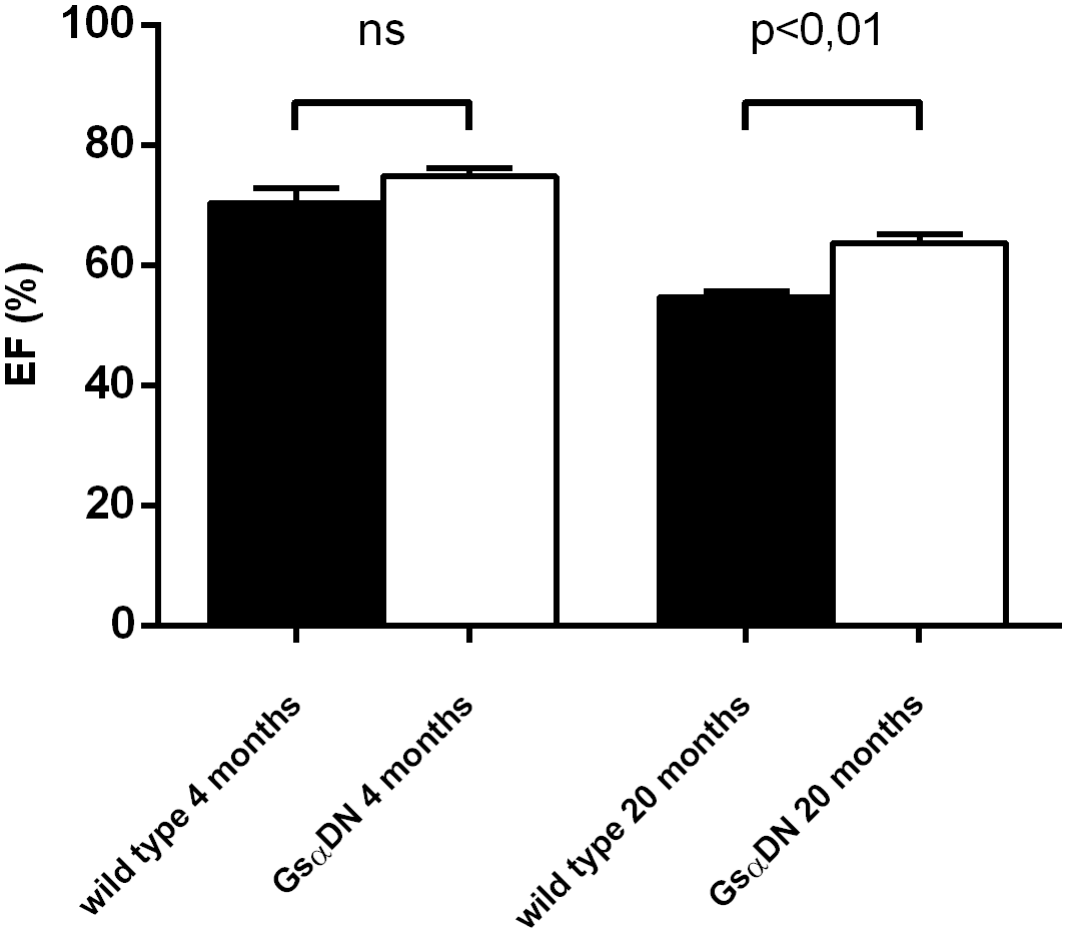
Ejection fraction (EF) in echocardiographic measurements in mice 4 months of age and 20 months of age wild type and Gsα-DN-mice.

Heart rate remained reduced in conscious Gsα-DN-mice in all measurements from 4 up to 20 months in comparison to wild type (data not shown). The prospective longitudinal echocardiographic examination at the age of 4 and 20 months revealed a significant lower anterior and posterior wall thickness in old Gsα-DN-mice compared to old WT littermates. Additionally, EF was significantly better preserved in old Gsα-DN-mice at 20 months, while there were no significant differences at baseline evaluation at 4 months (Fig 7). Echocardiographic data at the age of 20 months is shown in Table 4.

**Table 4:** Echocardiographic parameters at the age of 20 months.

	Heart rate [bpm]	AWTD [mm]	EDD [mm]	PWTD [mm]	EF [%]	FS [%]
**Wild type** n = 6	471 ± 22	0.6 ± 0.01	4.9 ± 0.3	0.7 ± 0.02	55 ± 1.0	23 ± 0.6
**Gsα-DN** n = 6	464 ± 18	0.5 ± 0.01	5.3 ± 0.2	0.5 ± 0.01	64 ± 1.5	29 ± 1.0
***p-value***	*n*.*s*.	*p<0*.*001*	*n*.*s*.	*p<0*.*001*	*p<0*.*001*	*p<0*.*01*

AWTD = anterior wall thickness diameter; EDD = end-diastolic diameter; PWTD = posterior wall thickness diameter; EF = ejection fraction; FS = fractional shortening.

Tissue samples of left ventricle after natural death showed no significant differences in the extent of collagen deposition in wild type and transgenic animals (5.3 ± 0.5 vs. 6.7 ± 0.6% collagen area fraction).

### Gsα-DN-mice show normal lifespan

**Fig 8:**
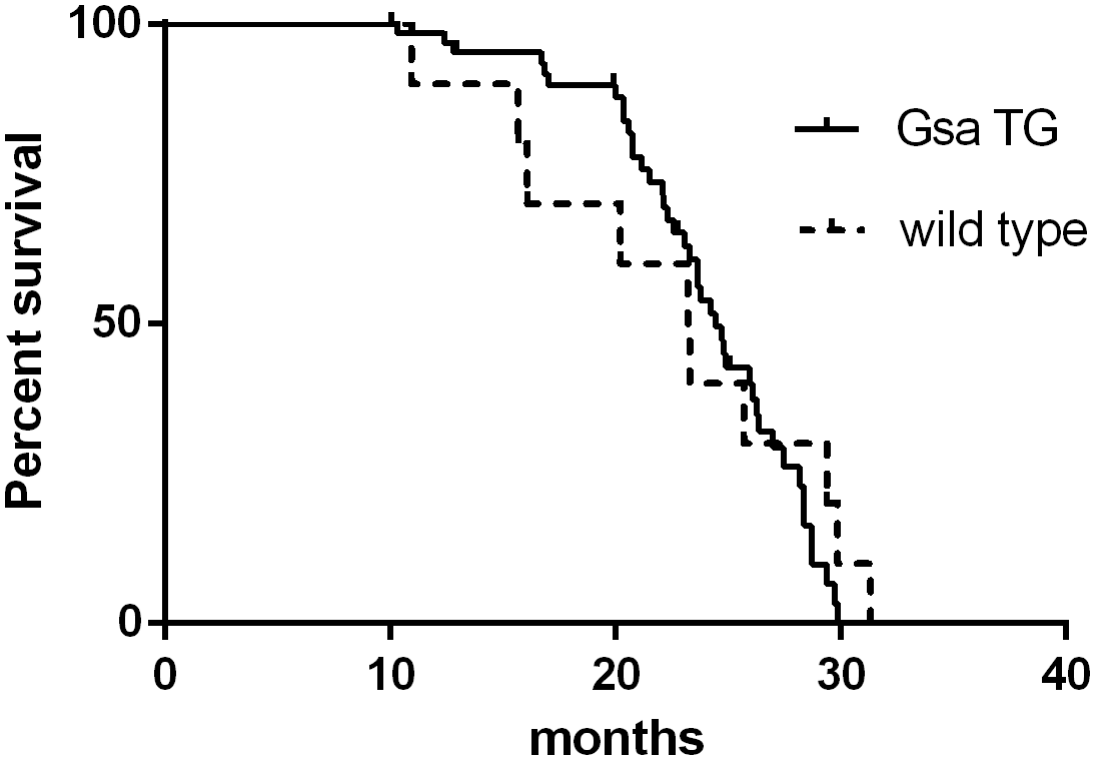
Kaplan-Meier survival analysis reveals no differences in survival rates between Gsα-DN-mice and wild type animals.

Gsα-DN-mice did not show higher mortality rates than WT littermates. There were no significant differences in the survival rates between the two groups (log rank test, p = 0.59; Fig 8). The median survival was 24 months in Gsα-DN-mice and 23 months in wild type animals. No differences in cause of death were observed between transgenic and wild type animals, especially not for the incidence of neoplasm or for pleural and pericardial effusion.

### Gsα-DN-mice show decreased β adrenergic responsiveness

**Fig 9:**
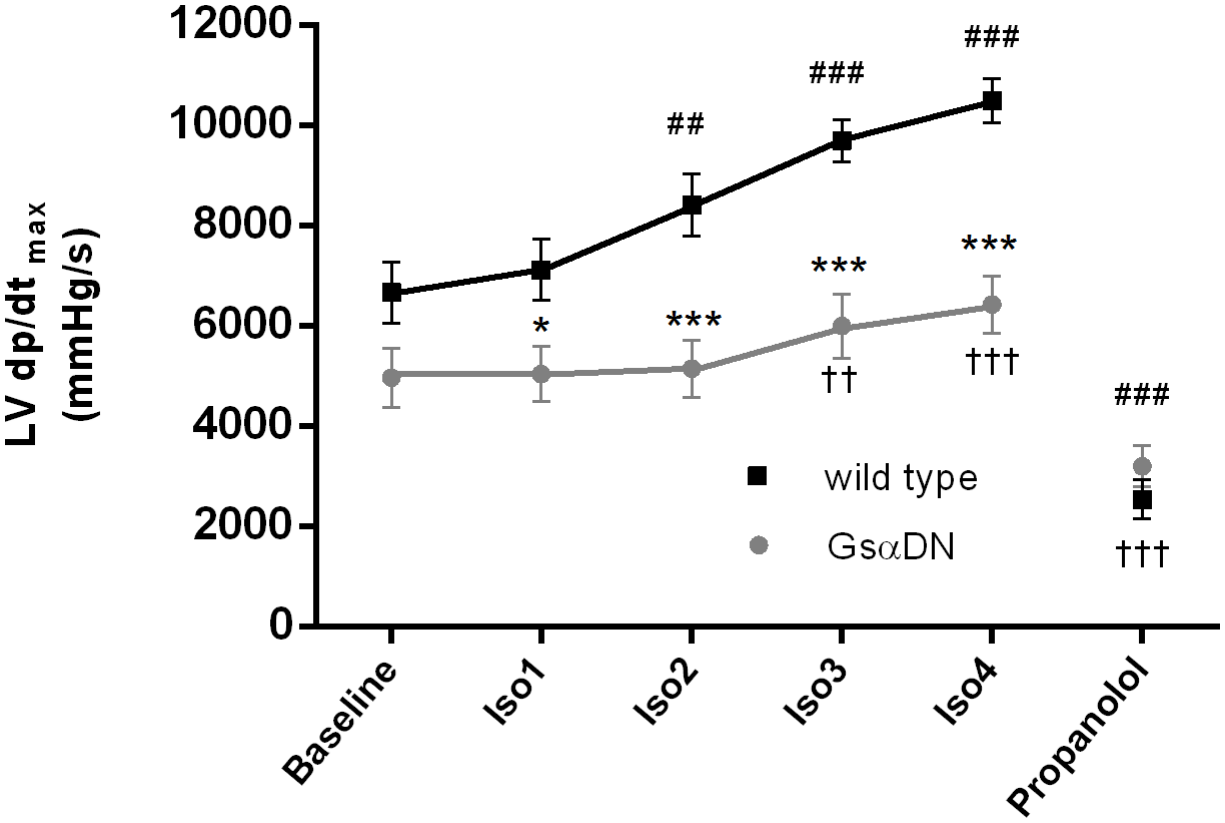
Gsα-DN-mice show decreased β-adrenergic responsiveness after acute adrenergic stimulation by intravenous infusion of isoproterenol (n = 12 for Gsα-DN-mice and n = 13 for wild type animals).

Acute β adrenergic stimulation by intravenous infusion of isoproterenol resulted in a significantly decreased β-adrenergic responsiveness in Gsα-DN-mice in comparison to WT littermates. Transgenic animals showed an increase in contractility only after higher doses of isoproterenol (0,1 μg/kg/min), while WT littermates responded already to small doses (0,02 μg/kg/min). Furthermore, the maximal increase in contractility was significantly higher in WT littermates compared to Gsα-DN-mice (+58% vs. +29%). β-blockade by propranolol revealed no baseline differences of hemodynamic parameters between wild type and Gsα-DN-mice (Fig 9).

To evaluate the extent of maximal β-adrenergic activability, transgenic animals were exposed to higher doses of isoproterenol (from 0,2 up to 2 μg/kg/min.). Maximal values of dp/dt_max_ were reached at an application rate of 0,5 μg/kg/min. (7605 ± 588 mmHg/s). Thus, β-adrenergic responsiveness in Gsα-DN-mice did not reach the extent of wild type responsiveness even under higher concentrations of isoproterenol.

### Gsα-DN-mice are protected against isoproterenol-induced hypertrophy

**Fig 10:**
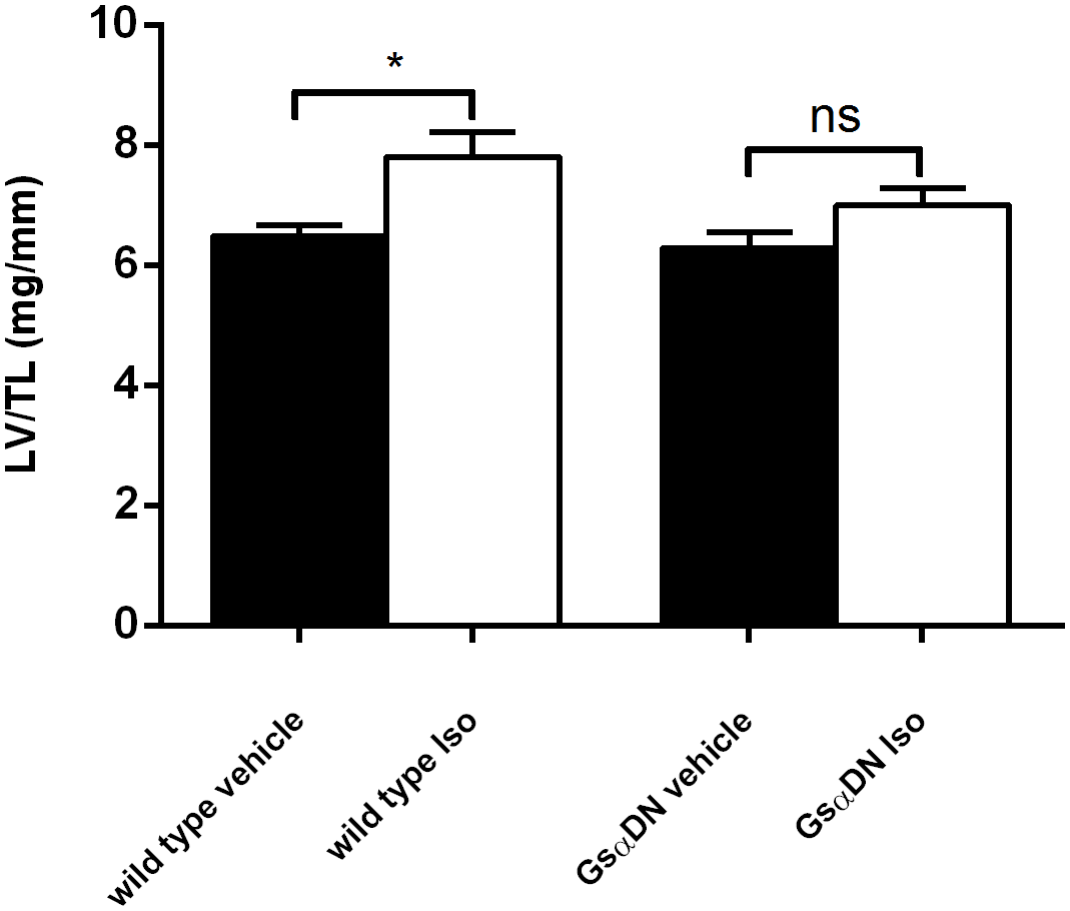
Gsα-DN-mice do not develop myocardial hypertrophy under chronic adrenergic stimulation. Increase of LV/TL following chronic subcutaneous administration of isoproterenol for 14 days (n = 7 for Gsα-DN-mice and n = 8 for wild type animals).

**Fig 11:**
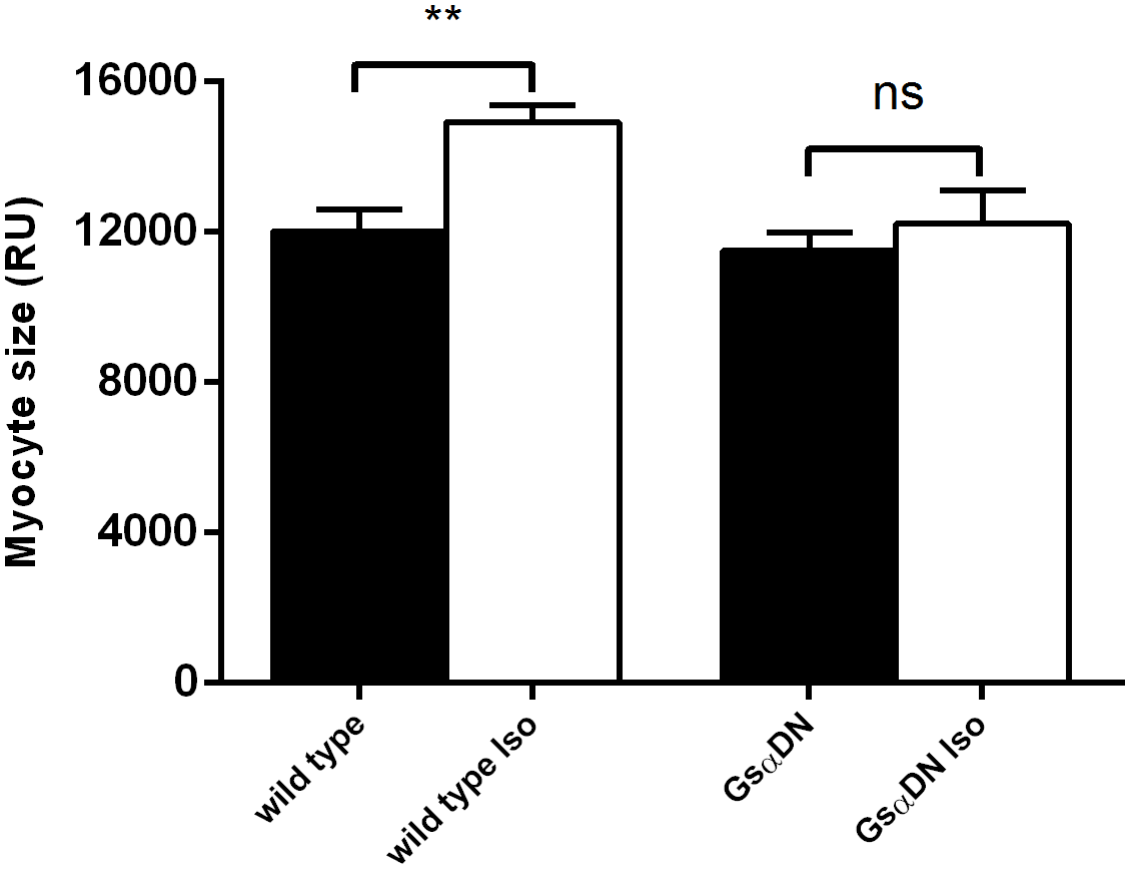
Increase of myocyte size following chronic subcutaneous administration of isoproterenol for 14 days (n = 7 for Gsα-DN-mice and n = 8 for wild type animals).

Under administration of intravenous isoproterenol for 14 days, heart rate was increasing in comparable extent both in wild types and in Gsα-DN-mice (679 ± 31 vs. 725 ± 28; n.s.). In comparison to isovolumic vehicle, wild type mice developed myocardial hypertrophy associated with an increase of LV to tibia length ratio (LV/TL) by 20% (7.8 ± 0.4 vs. 6.5 ± 0.2 mg/g, *p = 0*.*01*) and an increase of cardiac myocyte size by 24% (14927 ± 442 px vs. 12013 ± 583 px, *p<0*.*001*). In contrast, both parameters were not significantly elevated in Gsα-DN-mice as compared to vehicle (LV/TL 7.0 ± 0.3 mg/g vs. 6.3 ± 0.3 mg/g, *n*.*s*.; myocyte size 12217 ± 879 vs. 11494 ± 489, *n*.*s*.) (Figs 10 and 11).

After 14 days of chronic adrenergic stimulation, wild type mice develop a significant increase in both AWTD and PWTD. In contrast, wall thickness does not change in Gsα-DN-mice after isoproterenol stimulation. Echocardiographic parameters of systolic heart function were not different between Gsα-DN-mice and wild type after isoproterenol stimulation (data not shown).

**Fig 12:**
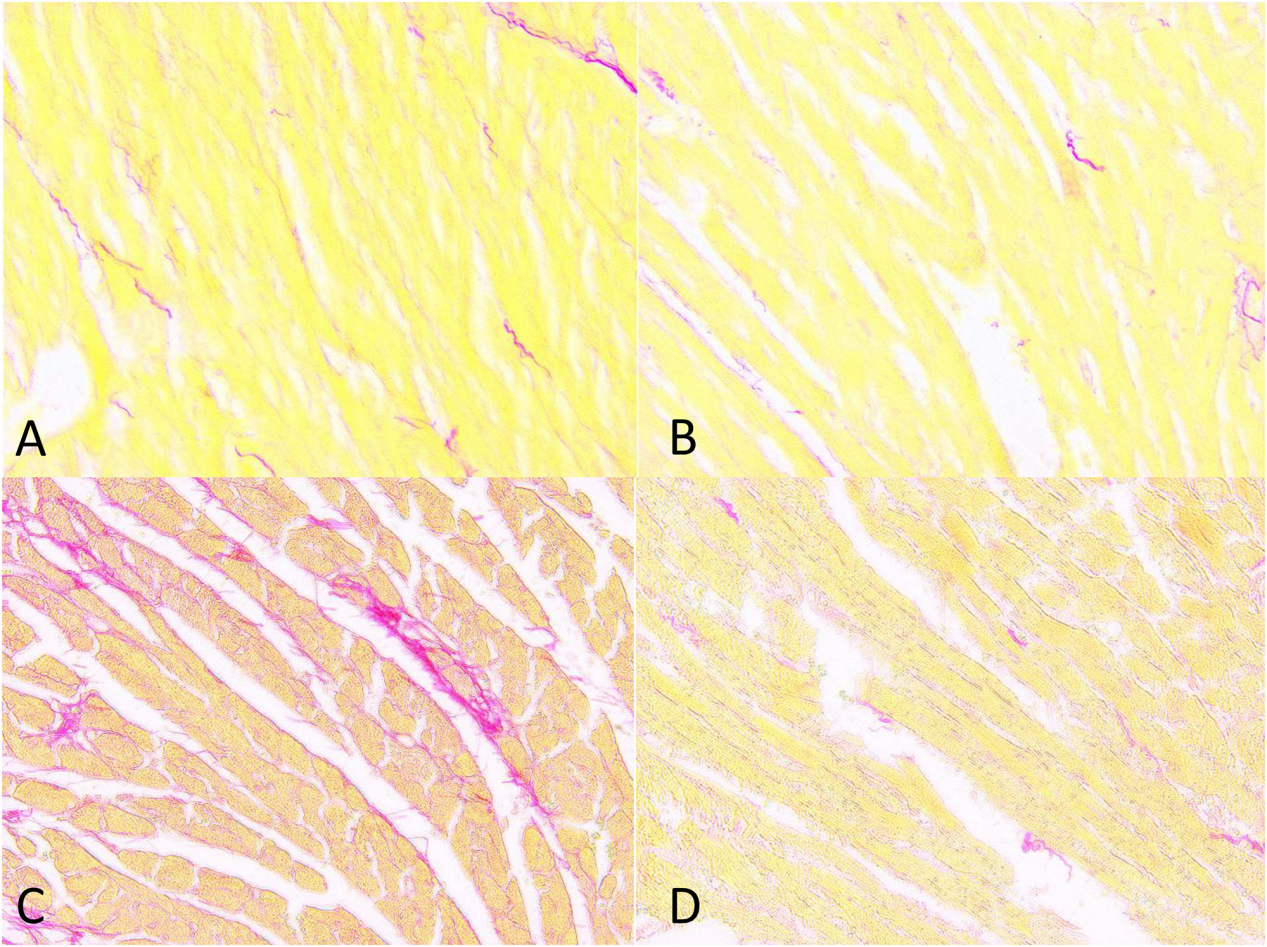
Gsα-DN-mice develop less collagen deposition after chronic adrenergic stimulation. **(A)** Exemplary picrosirius red staining of LV in wild type animals at 3 months; **(B)** in Gsα-DN-mice at 3 months; **(C)** in wild types after isoproterenol for 14 days; **(D)** in Gsα-DN-mice after isoproterenol for 14 days.

**Fig 13:**
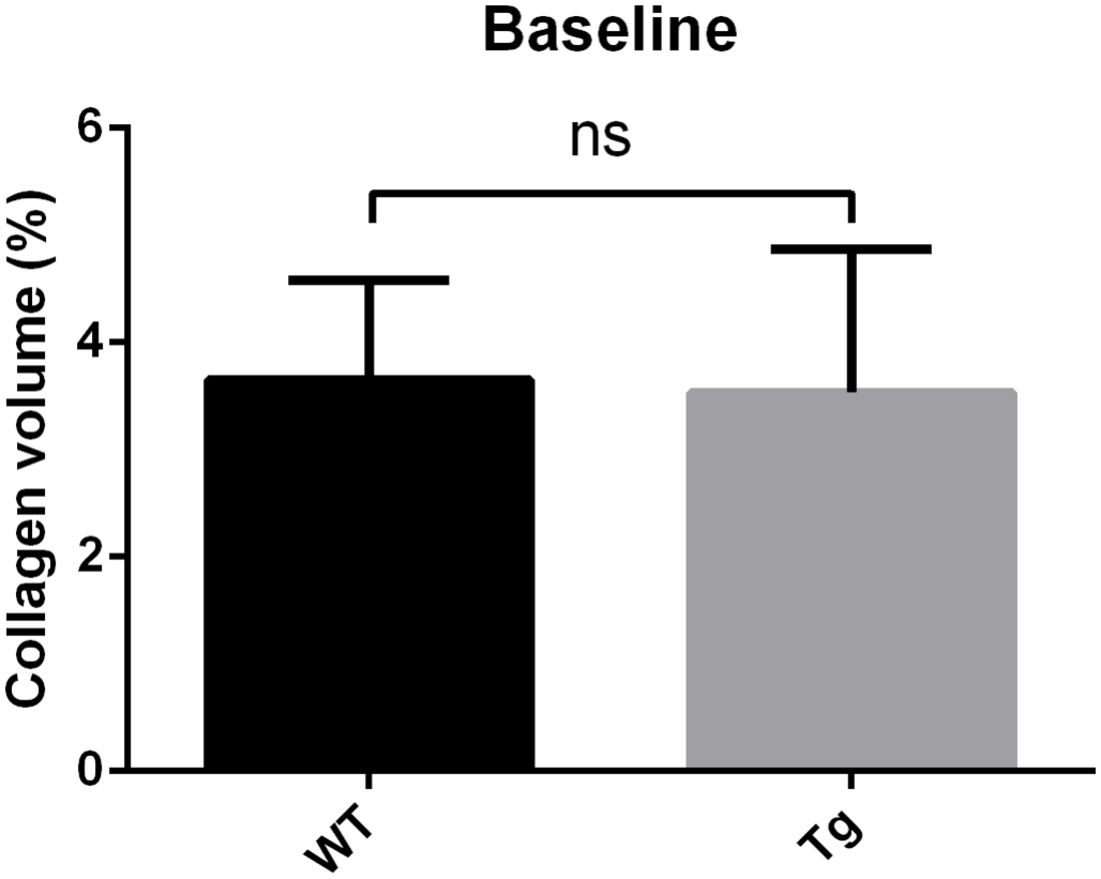
Regarding cardiac fibrosis, there were no significant differences in the extent of collagen deposition in wild type and transgenic animals at the age of 3 months.

**Fig 14:**
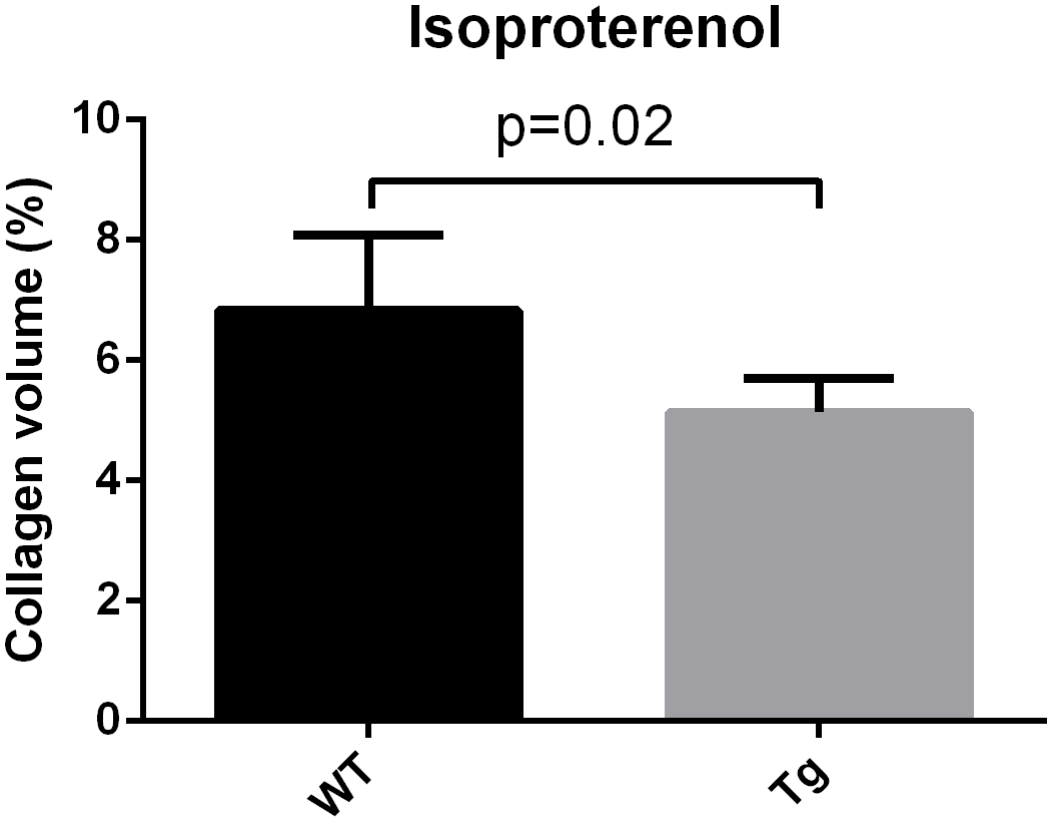
Gsα-DN-mice showed significantly less collagen deposition after chronic adrenergic stimulation in comparison to wild types.

Regarding cardiac fibrosis, Gsα-DN-mice showed significantly less collagen deposition after chronic adrenergic stimulation in comparison to wild types (5.1 ± 0.3 vs. 6.8 ± 0.5% collagen area fraction, *p<0*.*05*) (Figs 12, 13 and 14).

## Discussion

The present study demonstrates that cardiac selective overexpression of a dominant negative Gsα mutant leads to a viable phenotype and a reduction in cardiac β-adrenergic sensitivity with reduced maximal contractile response to acute β-adrenergic stimulation. Furthermore, Gsα-DN-mice are less prone to against detrimental structural changes (hypertrophy, dilatation, fibrosis) under chronic β-adrenergic stimulation. These alterations were associated with lower steady-state intracellular cAMP levels as compared to WT animals. The survival rate of Gsα-DN-mice was similar to that of wild-type mice. These data indicate that the selective inhibition of receptor-mediated stimulation of adenylyl cyclase by cardiac selective overexpression of a dominant negative Gsα mutant is not only compatible with a normal lifespan and cardiac phenotype, but, under conditions of chronic increased adrenergic drive, exerts cardioprotective effects.

Conversely, mice with heart-specific overexpression of Gsα show enhanced inotropy and chronotropy in response to acute β-adrenergic stimulation in young animals [18]. However, this murine model developed cardiomyopathy at older age, characterized by left ventricular (LV) dilatation and hypertrophy, reduced LV function, fibrosis, apoptosis, and shows an increased mortality [18–21]. In this model chronic blockade of β-adrenergic signaling by propranolol treatment prevented the development of cardiomyopathy and significantly reduced mortality [21, 22].

Altered β-adrenergic signaling predominantly with desensitization and blunted adrenergic agonist effects on contractile performance is a hallmark of the failing heart. A characteristic set of molecular alterations in components of the β-adrenergic signaling pathway has been identified [8]. Interestingly, in animal heart failure models such as mice bearing myosin heavy chain mutations that mimic familial hypertrophic cardiomyopathy (FHC) or dogs with pressure-overload left ventricular failure Gsα-protein levels were found to be reduced by 30–59% [11, 12]. In respect to therapies, it is critical whether these molecular alterations in β-adrenergic signaling in heart failure are the cause or consequence of heart failure. An important finding in this context is that targeted overexpression of Gsα accelerates heart failure in the FHC mouse model [11]. The results of previous studies in transgenic mice with overexpression of β-ARs, Gsα or PKA have established the adverse effects of chronic adrenergic stimulation while down regulation of β-adrenergic signaling in heart failure predominantly appears as a compensatory, protective mechanism.

The benefits of β-adrenergic receptor blockers in heart failure are well established. However, it is not known whether all downstream effects of β-adrenergic receptor blockers are beneficial. New drugs that selectively target downstream elements of the β-adrenergic signaling pathway may provide novel options for heart failure therapy.

The effect of chronic inhibition of β-adrenergic signaling has previously been examined in a few specific loss-of-function models downstream of cell surface receptors. Apart from the β_1_-and β_2-_ adrenoceptor knockout mice [23] two genetically engineered mice exist, in which stimulating components of β-adrenergic signaling were inhibited. Okumura et al. generated a mouse model with adenylyl cyclase type 5 knockout (AC5-KO) resulting in 30–40% decreased cAMP-PKA signaling [9, 24]. Unexpectedly, AC5 KO mice showed a significantly increased median lifespan compared to control littermates, likely due to protection from age related remodeling processes [25]. The cardiac phenotype and contractile performance at baseline was normal, while the response to β-adrenergic stimulation was decreased. AC5 KO mice were protected from heart failure after TAC and after long-term β-adrenergic stimulation [9, 26]. The authors hypothesize that this cardioprotective effect may result from a desensitization of β-adrenergic signaling in the heart as AC5 is a major isoform of AC in the myocardium [25].

El-Armouche et al. created a loss-of-function mouse model with phosphatase inhibitor-1 knockout (I-1-KO) [10]. I-1-deficient mice show normal heart structure and mildly reduced sensitivity but unchanged maximal contractile response to β-adrenergic stimulation. However, similarly to AC5-KO mice, they were partially protected from hypertrophy and dilatation after chronic β-adrenergic stimulation.

Similar to the AC5-KO [24], Gsα-DN mice did not show reduced LV function at baseline. In the Gsα-DN mice β-adrenergic responsiveness was significanty reduced associated with a better preservation of cardiac function with aging and protection against isoproterenol-induced hypertrophy. In contrast to AC5-KO mice, Gsα-DN-mice were not characterized by an significantly increased lifespan.

Results from our study suggest that diminishing Gsα in the heart might play a critical role in transducing β-adrenergic receptor signaling and coupling the high-affinity receptor to adenylyl cyclase such that at any given level of receptor occupancy there is reduced cAMP-generation and therefore each sympathetic stimulus results in lower cAMP signal and, consequently, reduced myocardial contractility and heart rate.

Gsα-DN mice have an intracellular cAMP reduction of 50% in comparison to wild type animals. Iiri et al. showed a strong dominant negative activity with up to 80% reduction in Gs-dependent cAMP accumulation in COS-7 cells for the triple mutant used in this transgenic mouse model [16]. The authors propose that this mutant most likely acts by sequestering receptors. This mode of action is conceivable as most cells contain many more copies of G proteins than of the receptor that stimulate it; the ratio of G protein to receptor maybe as high as 10:1 [27]. Furthermore, a variety of data have previously shown that the rate of exchange of GDP for GTP at the G protein is rate limiting in the activation of adenelyl cyclase, as shown in the equation Gsα-GDP + GTP → Gsα-GTP+GDP [19]. Thus a relative small change in the content of Gsα can impact on receptor- Gsα-coupling and the rate of adenelyl cyclase activation in the heart.

Future studies might evaluate how downstream targets of cAMP signaling like phosphorylation of phospholamban are affected by overexpression of a dominant negative Gsα mutant [28] and clarify if changed Gα i2 and Gαi3 protein levels play a role in the functional effects observed in this model. Our animal model might be helpful in dissecting other signaling pathways such as the β-arrestin-dependent signaling. At this time there is no strongly biased ligand at the β1-AR which blocks G protein-dependent signaling while stimulating β-arrestin-dependent signaling, which might be cardioprotective [14].

As the use of beta-blockade has been proven to be of benefit in clinical practice, it is conceivable that interrupting distal components in the beta-adrenergic receptor-G protein-adenylyl cyclase pathway may also provide targets for future therapeutic modalities for heart failure. In theory, it may be desirable to block receptor signaling via one type of G protein but not via the other G proteins or β-arrestin-dependent signaling (resulting in biased receptor signaling). This might be achieved by compounds that bind selectively to individual G proteins, as it has been shown for several suramin analogons like NF503 or NF449 [29]. More selective inhibition of downstream elements of the beta-adrenergic signaling might have fewer side effects compared to classic beta-blockade therapy.

In conclusion, overexpression of a dominant negative mutant of Gsα leads to decreased β-adrenergic responsiveness and is protective against isoproterenol-induced hypertrophy. Gsα-DN-mice are a suitable model for characterization of β-adrenergic signal transduction in heart failure and for evaluation of potential therapeutic targets for prevention of maladaptive remodeling.

## Supporting Information

S1 ARRIVE Checklist The ARRIVE Guidelines Checklist. (PDF)

## References

[1] Eschenhagen T. Beta-adrenergic signaling in heart failure-adapt or die. Nat Med. 2008;14(5):485–7. Epub 2008/05/09. nm0508-485 [pii] 10.1038/nm0508-485 . 18463653

[2] MERIT-HF Study Group. Effect of metoprolol CR/XL in chronic heart failure: Metoprolol CR/XL Randomised Intervention Trial in Congestive Heart Failure (MERIT-HF). Lancet. 1999;353(9169):2001–7. Epub 1999/06/22. S0140673699044402 [pii]. . 10376614

[3] Hjalmarson A, Goldstein S, Fagerberg B, Wedel H, Waagstein F, Kjekshus J, et al Effects of controlled-release metoprolol on total mortality, hospitalizations, and well-being in patients with heart failure: the Metoprolol CR/XL Randomized Intervention Trial in congestive heart failure (MERIT-HF). MERIT-HF Study Group. JAMA. 2000;283(10):1295–302. Epub 2000/03/14. joc92053 [pii]. . 10714728 10.1001/jama.283.10.1295

[4] Krum H, Roecker EB, Mohacsi P, Rouleau JL, Tendera M, Coats AJ, et al Effects of initiating carvedilol in patients with severe chronic heart failure: results from the COPERNICUS Study. JAMA. 2003;289(6):712–8. Epub 2003/02/15. joc21658 [pii]. . 12585949 10.1001/jama.289.6.712

[5] CIBIS-II Investigators and Committees. The Cardiac Insufficiency Bisoprolol Study II (CIBIS-II): a randomised trial. Lancet. 1999;353(9146):9–13. Epub 1999/02/19. S0140673698111819 [pii]. . 10023943

[6] Packer M, Bristow MR, Cohn JN, Colucci WS, Fowler MB, Gilbert EM, et al The effect of carvedilol on morbidity and mortality in patients with chronic heart failure. U.S. Carvedilol Heart Failure Study Group. N Engl J Med. 1996;334(21):1349–55. Epub 1996/05/23. 10.1056/NEJM199605233342101 . 8614419

[7] Neubauer S. The failing heart—an engine out of fuel. N Engl J Med. 2007;356(11):1140–51. Epub 2007/03/16. 17360992 10.1056/NEJMra063052

[8] El-Armouche A, Eschenhagen T. Beta-adrenergic stimulation and myocardial function in the failing heart. Heart Fail Rev. 2009;14(4):225–41. Epub 2008/12/30. 19110970 10.1007/s10741-008-9132-8

[9] Okumura S, Takagi G, Kawabe J, Yang G, Lee MC, Hong C, et al Disruption of type 5 adenylyl cyclase gene preserves cardiac function against pressure overload. Proc Natl Acad Sci U S A. 2003;100(17):9986–90. Epub 2003/08/09. PubMed Central PMCID: PMC187910. 12904575 10.1073/pnas.1733772100 PMC187910

[10] El-Armouche A, Wittkopper K, Degenhardt F, Weinberger F, Didie M, Melnychenko I, et al Phosphatase inhibitor-1-deficient mice are protected from catecholamine-induced arrhythmias and myocardial hypertrophy. Cardiovasc Res. 2008;80(3):396–406. Epub 2008/08/12. cvn208 [pii] 10.1093/cvr/cvn208 . 18689792

[11] Hardt SE, Geng YJ, Montagne O, Asai K, Hong C, Yang GP, et al Accelerated cardiomyopathy in mice with overexpression of cardiac G(s)alpha and a missense mutation in the alpha-myosin heavy chain. Circulation. 2002;105(5):614–20. Epub 2002/02/06. . 11827928 10.1161/hc0502.103012

[12] Longabaugh JP, Vatner DE, Vatner SF, Homcy CJ. Decreased stimulatory guanosine triphosphate binding protein in dogs with pressure-overload left ventricular failure. J Clin Invest. 1988;81(2):420–4. Epub 1988/02/01. 10.1172/JCI113335 3123520 PMC329585

[13] Noma T, Lemaire A, Naga Prasad SV, Barki-Harrington L, Tilley DG, Chen J, et al Beta-arrestin-mediated beta1-adrenergic receptor transactivation of the EGFR confers cardioprotection. J Clin Invest. 2007;117(9):2445–58. Epub 2007/09/06. 10.1172/JCI31901 17786238 PMC1952636

[14] Tilley DG. G protein-dependent and G protein-independent signaling pathways and their impact on cardiac function. Circ Res. 2011;109(2):217–30. Epub 2011/07/09. 10.1161/CIRCRESAHA.110.231225 21737817 PMC3138127

[15] Yu S, Yu D, Lee E, Eckhaus M, Lee R, Corria Z, et al Variable and tissue-specific hormone resistance in heterotrimeric Gs protein alpha-subunit (Gsalpha) knockout mice is due to tissue-specific imprinting of the gsalpha gene. Proc Natl Acad Sci U S A. 1998;95(15):8715–20. Epub 1998/07/22. 9671744 10.1073/pnas.95.15.8715 PMC21142

[16] Iiri T, Bell SM, Baranski TJ, Fujita T, Bourne HR. A Gsalpha mutant designed to inhibit receptor signaling through Gs. Proc Natl Acad Sci U S A. 1999;96(2):499–504. Epub 1999/01/20. 9892662 10.1073/pnas.96.2.499 PMC15165

[17] Iwase M, Bishop SP, Uechi M, Vatner DE, Shannon RP, Kudej RK, et al Adverse effects of chronic endogenous sympathetic drive induced by cardiac GS alpha overexpression. Circ Res. 1996;78(4):517–24. Epub 1996/04/01. . 8635208 10.1161/01.res.78.4.517

[18] Iwase M, Uechi M, Vatner DE, Asai K, Shannon RP, Kudej RK, et al Cardiomyopathy induced by cardiac Gs alpha overexpression. Am J Physiol. 1997;272(1 Pt 2):H585–9. Epub 1997/01/01. . 9038982 10.1152/ajpheart.1997.272.1.H585

[19] Gaudin C, Ishikawa Y, Wight DC, Mahdavi V, Nadal-Ginard B, Wagner TE, et al Overexpression of Gs alpha protein in the hearts of transgenic mice. J Clin Invest. 1995;95(4):1676–83. Epub 1995/04/01. 10.1172/JCI117843 7706476 PMC295676

[20] Geng YJ, Ishikawa Y, Vatner DE, Wagner TE, Bishop SP, Vatner SF, et al Apoptosis of cardiac myocytes in Gsalpha transgenic mice. Circ Res. 1999;84(1):34–42. Epub 1999/01/23. . 9915772 10.1161/01.res.84.1.34

[21] Asai K, Yang GP, Geng YJ, Takagi G, Bishop S, Ishikawa Y, et al Beta-adrenergic receptor blockade arrests myocyte damage and preserves cardiac function in the transgenic G(salpha) mouse. J Clin Invest. 1999;104(5):551–8. Epub 1999/09/16. 10.1172/JCI7418 10487769 PMC408547

[22] Ho D, Yan L, Iwatsubo K, Vatner DE, Vatner SF. Modulation of beta-adrenergic receptor signaling in heart failure and longevity: targeting adenylyl cyclase type 5. Heart Fail Rev. 2010;15(5):495–512. Epub 2010/07/27. 10.1007/s10741-010-9183-5 . 20658186 PMC3655553

[23] Lee S, Schwinger RH, Brixius K. Genetically changed mice with chronic deficiency or overexpression of the beta-adrenoceptors—what can we learn for the therapy of heart failure? Pflugers Arch. 2008;455(5):767–74. Epub 2007/09/18. 10.1007/s00424-007-0324-1 . 17874127

[24] Okumura S, Kawabe J, Yatani A, Takagi G, Lee MC, Hong C, et al Type 5 adenylyl cyclase disruption alters not only sympathetic but also parasympathetic and calcium-mediated cardiac regulation. Circ Res. 2003;93(4):364–71. Epub 2003/07/19. 12869393 10.1161/01.RES.0000086986.35568.63

[25] Yan L, Vatner DE, O'Connor JP, Ivessa A, Ge H, Chen W, et al Type 5 adenylyl cyclase disruption increases longevity and protects against stress. Cell. 2007;130(2):247–58. Epub 2007/07/31. S0092-8674(07)00677-0 [pii] 10.1016/j.cell.2007.05.038 . 17662940

[26] Okumura S, Vatner DE, Kurotani R, Bai Y, Gao S, Yuan Z, et al Disruption of type 5 adenylyl cyclase enhances desensitization of cyclic adenosine monophosphate signal and increases Akt signal with chronic catecholamine stress. Circulation. 2007;116(16):1776–83. Epub 2007/09/26. CIRCULATIONAHA.107.698662 [pii] 10.1161/CIRCULATIONAHA.107.698662 . 17893275

[27] Lustig KD, Conklin BR, Herzmark P, Taussig R, Bourne HR. Type II adenylylcyclase integrates coincident signals from Gs, Gi, and Gq. J Biol Chem. 1993;268(19):13900–5. Epub 1993/07/05. . 8390980

[28] Kim SJ, Yatani A, Vatner DE, Yamamoto S, Ishikawa Y, Wagner TE, et al Differential regulation of inotropy and lusitropy in overexpressed Gsalpha myocytes through cAMP and Ca2+ channel pathways. J Clin Invest. 1999;103(7):1089–97. 10.1172/JCI4848 10194482 PMC408254

[29] Hohenegger M, Waldhoer M, Beindl W, Boing B, Kreimeyer A, Nickel P, et al Gsalpha-selective G protein antagonists. Proc Natl Acad Sci U S A. 1998;95(1):346–51. 9419378 10.1073/pnas.95.1.346 PMC18220

